# A Review on the Progress and Strategies of *Helicobacter pylori* Eradication Therapy for Patients With Penicillin Allergy

**DOI:** 10.1155/grp/5587248

**Published:** 2026-04-08

**Authors:** Yuxin Zhang, Baojun Suo, Xueli Tian, Cailing Li, Xinlu Ren, Kai Zhou, Changmin Mi, Yuxin Wang, Lingling Ma, Liya Zhou, Zhiqiang Song

**Affiliations:** ^1^ Department of Gastroenterology, Peking University Third Hospital, Beijing, China, puh3.net.cn

**Keywords:** bismuth quadruple therapy, eradication therapy, *Helicobacter pylori*, new regimen, penicillin allergy

## Abstract

**Background:**

*Helicobacter pylori (H. pylori)* eradication in penicillin‐allergic patients poses a challenge. The currently recommended regimens are inadequate for clinical eradication therapy. Recent years have seen reports of new drugs, regimens, and study evidence in this field; yet comprehensive review articles on the treatment of *H. pylori* infection in penicillin‐allergic patients are scarce.

**Methods:**

A literature search of PubMed, Web of Science, Embase, China National Knowledge Infrastructure, the China Science and Technology Journal Database, and the Wanfang Database up to January 10, 2026, was conducted with the search strategy (“*Helicobacter pylori”* OR *“H. pylori*”) AND (“treatment” OR “therapy”) AND (“penicillin” OR “beta‐lactam”) AND (“allergy”, “allergic” OR “anaphylaxis”) for both English and Chinese language publications

**Results:**

The final literature review included 132 treatment groups from 56 studies in nine different countries or regions, with a total of 5542 patients included in the analyses. The overall eradication rates of triple regimens were unsatisfactory (72.9%, 95% CI 70.7%–75.2%). The classic quadruple therapy (PPI, bismuth, tetracycline, and metronidazole) is commonly recommended due to its relatively satisfactory efficacy and sufficient research evidence (81.4%, 95% CI 78.6%–84.2%). The mean eradication rate for other regimens containing new drugs such as minocycline, cefuroxime, sitafloxacin, vonoprazan, and rifabutin is well (> 80%).

**Conclusion:**

The classical bismuth quadruple therapy used to be the most commonly used regimen. The overall eradication efficacy of triple therapies is unacceptable. Although new drugs and regimens have emerged, their efficacy needs confirmation through more high‐quality, large‐scale clinical studies to identify the optimal eradication therapy regimen. Individualized treatment, particularly therapies tailored based on the results of genetic antibiotic resistance testing, represents an important direction for future research.

## 1. Introduction

*Helicobacter pylori (H. pylori)* infection is a significant global public health concern. The World Health Organization reports that approximately 50% of the world′s population is infected, with rates reaching around 70% in developing countries [1]. The presence of *H. pylori* is closely associated with the pathogenesis of gastritis, peptic ulcer disease, lymphoma of gastric mucosa‐associated lymphoid tissues, and gastric carcinoma [2]. Eradicating *H. pylori* is crucial for treating or preventing these diseases [3], making *H. pylori* eradication a significant clinical need.

The successful eradication of *H. pylori* infection is becoming increasingly challenging. Commonly used eradication regimens are losing effectiveness due to the rising antibiotic resistance, particularly to clarithromycin, levofloxacin, and metronidazole, and the limited availability of drugs such as tetracycline, furazolidone, and bismuth in clinical practice [4–8]. Amoxicillin is a key component in *H. pylori* eradication regimens due to its low resistance rate, safety, affordability, and widespread availability.

However, approximately 6%–25% of the population cannot use amoxicillin due to penicillin allergy [9], complicating *H. pylori* eradication. To address these challenges, global recommendations have been made based on limited evidence and local conditions (see Table 1). The Maastricht VI/Florence consensus [2] recommends the proton pump inhibitor (PPI)–clarithromycin–metronidazole triple regimen as first‐line therapy in regions with low clarithromycin resistance and bismuth quadruple therapy (BQT) (PPI–bismuth–tetracycline–metronidazole) as first‐line therapy in regions with high clarithromycin resistance (> 15%). For second‐line treatment, BQT is recommended if not previously prescribed, with a fluoroquinolone‐containing regimen also recommended if local *H. pylori* isolates are quinolone‐sensitive. The American College of Gastroenterology (ACG) guidelines [10] recommend tailored drug and regimen selection based on local resistance patterns, previous eradication regimens, and macrolide and quinolone exposure history. The Chinese eradication guidelines [11] suggest using the second‐generation cephalosporin cefuroxime instead of amoxicillin for eradication in penicillin‐allergic patients. The Japanese eradication guidelines [12] recommend specific drug regimens based on clarithromycin resistance rates, with sitafloxacin‐containing and minocycline‐containing regimens considered for patients with drug‐resistant *H. pylori*.

**Table 1:** Recommended *Helicobacter pylori* eradication regimens from major guidelines and consensus in penicillin‐allergic patients.

Guideline and consensus	Year	Therapy
Maastricht VI/Florence	2022	First‐line: Low clarithromycin resistance regions: PPI + C + M; high clarithromycin resistance regions: PPI + B + T + M Rescue: PPI + B + T + M (if not previously prescribed) or fluoroquinolone‐containing regimen
United States of America (ACG)	2017	First‐line: Low clarithromycin resistance regions without macrolide exposure: PPI + C + M or PPI + B + T + M; high clarithromycin resistance regions or with macrolide exposure: PPI + B + T + M Rescue: patients who received PPI + C + M : PPI + B + T + M; patients who received PPI + B + T + M without quinolone exposure: PPI + L + M or PPI + C + M; patients who received PPI + B + T + M with quinolone exposure: PPI + B + C + M (high‐dose PPI + M)
World Gastroenterology Organization (WGO)	2023	PPI + C + M or PPI + B + T + M
Association of Southeast Asian Nations (ASEAN) countries	2017	PPI + B + T + M or therapy depends on the local pattern of susceptibility
Canada	2017	First‐line: PPI + B + T + M
China (Mainland)	2022	PPI + B + T + M or PPI + B + L + Ce or PPI + B + C + M (full dose)
China (Taiwan)	2017	PPI + B + T + M or PPI + C + M
China (Hongkong)	2023	First‐line: Low clarithromycin resistance regions: PPI + C + M; High clarithromycin resistance regions: PPI + B + T + M
Korea	2020	First‐line: PPI + B + T + M
Japan	2019	Without clarithromycin resistance: PPI + C + M; with resistant bacteria or unknown: PPI + Si + M or PPI + Mi + M
Spain	2021	First‐line: PPI + B + T + M Rescue: patients who received PPI + C + M : PPI + B + T + M; patients who received PPI + B + T + M : PPI + B + L + C
Italy	2015	First‐line: PPI + C + M

Abbreviations: B, bismuth; C, clarithromycin; L, levofloxacin; M, metronidazole; Mi, minocycline; PPI, proton pump inhibitor; Si, sitafloxacin; T, tetracycline.

There is a paucity of comprehensive literature reviews and analysis articles on *H. pylori* eradication in penicillin‐allergic patients. New drugs and eradication regimens have been introduced in recent years, yet the existing recommended regimens are not optimal for meeting the eradication needs of these patients. Therefore, this review is aimed at comprehensively reviewing and analyzing published studies on *H. pylori* eradication in penicillin‐allergic patients, offering feasible and improved options to enhance the efficacy of *H. pylori* eradication in this significant patient subgroup.

## 2. Method

### 2.1. Literature Search

A systematic literature search was conducted in PubMed, Web of Science, Embase, China National Knowledge Infrastructure, the China Science and Technology Journal Database, and the Wanfang Database up to January 10, 2026, using the search strategy (“Helicobacter pylori” OR “H pylori”) AND (“treatment” OR “therapy”) AND (“penicillin” OR “beta‐lactam”) AND (“allergy” OR “allergic” OR “anaphylaxis”) for both English and Chinese language publications. Reference lists from the papers selected in the electronic search were hand‐searched to identify further relevant studies.

Data extracted from each study included region, study type (single‐center or multicenter, prospective or retrospective), sample size, patient type (first‐line or salvage), treatment regimen, duration of therapy, eradication rate, adverse event rate, and adherence (see Tables 2 and 3). Eradication rates by intention‐to‐treat (ITT) and per‐protocol analyses were the primary endpoints considered. Continuous variables are presented as the arithmetic mean and respective standard deviation. Qualitative variables are presented as percentages and 95% confidence intervals (95% CI). In instances where comprehensive estimates were presented for a given regimen category, the descriptive summaries were employed, with the relevant binomial confidence intervals being utilized to substantiate these calculations.

**Table 2:** Triple therapy for *Helicobacter pylori* eradication in patients allergic to penicillin.

Author	Year	Country/region	Center	Study design	Sample size	Patient type	Treatment	Duration (days)	Eradication rate (ITT analysis, %)	Eradication rate (PP analysis, %)	Adverse reactions (%)	Compliance (%)
Gisbert JP et al.[13]	2005	Spain	S	P	12	1st line	PPI + C + M	7	58	64	17	92
			M		9	3rd line	PPI + C + Rf	10	11	17	89	67
			S		2	4th line	PPI + M + L	10	100	100	50	100

Rodríguez‐Torres M et al.[14]	2005	Puerto Rico	S	R	17	1st line	PPI + M + T	10	85	/	/	/
					3	2nd line	PPI + M + T	10	100	/	/	/

Matsushima M et al.[15]	2006	Japan	S	R	5	1st line	PPI + T + M	7‐14	80	100	/	80

Konno T et al.[16]	2010	Japan	S	R	1	1st line	PPI + L + Mi	14	100	/	/	/

Gisbert JP et al.[17]	2010	Spain	M	P	50	1st line	PPI + C + M	7	54	55	10	98
					15	2nd line	PPI + C + L	10	73	73	20	100

Siala N et al.[18]	2010	Tunisia	S	R	1	1st line	PPI + C + M	10	0	/	/	/

Sahara S,et al.[19]	2013	Japan	S	P	4	1st line	PPI + M + Si	7	100	/	10	/
					5	2nd line	PPI + M + Si	7	100	/	10	/

Furuta T et al.[20]	2014	Japan	S	R	11	1st line	PPI + M + Si	7‐14	100	/	64	/
					12	2nd line	PPI + M + Si	7‐14	100	/	42	/
					5	3rd line	PPI + M + Si	7‐14	100	/	80	/

Tanaka A et al.[21]	2014	Japan	S	P	30	1st line	PPI + C + M	7	56.7	/	/	/
					11	2nd line	PPI + M + Si	7	81.8	/	/	/
					5	1st line	PPI + M + Si	7	80	/	/	/

Furuta T et al[22]	2015	Japan	S	P	44	ND	PPI + M + Si	7	97.7	/	/	/

Gisbert JP et al[23]	2015	Spain	M	P	112	1st line	PPI + C + M	7	57	59	14	94
					64	2nd line	PPI + C + L	10	64	71	24	91
					7	3rd line	PPI + C + Rf	10	14	20	71	71
					3	3rd line	PPI + C + L	10	33	50	67	67
					2	4th line	PPI + C + Rf	10	100	100	67	100
					2	4th line	PPI + C + L	10	50	0	100	100

Kataoka S[24]	2016	Japan	S	P	19	ND	V + M + Mi	14	100	/	/	/
					7	ND	PPI + M + Mi	14	100	/	/	/

Tanaka A et al.[25]	2016	Japan	ND	P	35	ND	PPI + C + M	7	57	/	/	/
					10	ND	V + M + Si	7	100	/	/	/
					10	ND	PPI + M + Si	7	80	/	/	/

Furuta T et al.[26]	2017	Japan	S	R	9	ND	V + C + M^a^	7	80	/	/	/
					25	ND	V + Si + M^a^	7	96	/	/	/
					4	ND	V + Mi + Si^a^	7	75	/	/	/

Nagahara A. et al.[27]	2017	Japan	S	P	1	1st line	V + Si + M	10	100	/	/	/
					12	1st line	V + C + M	7	91.7	/	/	/
					2	2nd line	V + Si + M	10	50	/	/	/
					3	2nd line	V + C + M	7	66.7	/	/	/

Tanaka A et al[28]	2017	Japan	ND	P	33	ND	V + M + Si	7	97	97	/	/

Mori H et al.[29]	2017	Japan	S	P	33	1st line	PPI + M + Si	10	100	/	/	100
					19	2nd line	PPI + M + Si	10	85	/	/	100
					5	3rd line	PPI + M + Si	10	40	/	/	100

Ono S et al.[30]	2017	Japan	S	R	14	1st line	V + M + Si	7	92.9	100	/	/
					13	1st line	V + C + M	7	92.3	92.3	/	/
					20	1st line	PPI + M + Si	7	100	100	/	/
					10	1st line	PPI + C + M	7	50.0	55.6	/	/
					3	2nd line	V + M + Si	7	66.7	66.7	/	/
					1	2nd line	V + C + M	7	100	100	/	/
					24	2nd line	PPI + M + Si	7	100	100	/	/
					3	2nd line	PPI + C + M	7	33.3	33.3	/	/

Osumi H et al[31]	2017	Japan	S	P	5	1st line	PPI + M + Mi	7	100	100	/	/

Sue S et al.[32]	2017	Japan	S	P	20	1st line	V + C + M	7	100	100	40	100
				R	30	1st line	PPI + C + M	7	83	83	20	64

Tanaka A et al.[33]	2017	Japan	ND	R	10	1st line	PPI + M + Si	7	80	/	/	/
					13	2nd line	PPI + M + Si	7	84.6	/	/	/

Long X et al.[34]	2018	China	S	P(RCT)	33	1st line	PPI + C + M	14	63.6	70.0	45.5	93.9

Zhang J et al.[35]	2019	China	S	R	25	1st line	PPI + C + M	7‐14	56	/	/	/

Luo L et al.[36]	2020	China	S	P	2	ND	PPI + T + M^a^	14	100	100	/	/
					1	ND	PPI + L + M^a^	14	100	100	/	/
					5	ND	PPI + C + M^a^	14	100	100	/	/

Masaoka T et al.[37]	2020	Japan	M	P	69	1st line	PPI + M + Si	10	92.6	/	/	/

Nyssen OP et al.[38]	2020	Europe	M	R	285	1st line	PPI + C + M	7‐14	69	69	23	98
					54	1st line	PPI + C + L	7‐14	80	82	19	98
					17	2nd line	PPI + M + L	7‐14	77	77	18	100
					20	2nd line	PPI + C + L	7‐14	75	74	21	87
					2	3rd line	PPI + C + L	7‐14	50	50	0	100

Sue S et al.[39]	2021	Japan	S	P	43	1st line	V + C + M	7	92.9	/	/	/
					17	2nd line	V + M + Si	7	88.2	88.2	25.0	100

Tepes B et al.[40]	2021	Slovenia	M	P	35	1st line	PPI + C + M	14	83	83	/	/

Adachi K et al.[41]	2023	Japan	S	R	10	ND	V + M + Si	7	90	90	20	/
					35	ND	V + C + M	7	94	100	9	/
					8	ND	PPI + C + M	7	50	50	13	/
Zhang CY [62]	2024	China	S	P	87	1st line	PPI+B+Ce+C	14	89.7	/	(14.9	/

Abbreviations: B, bismuth; C, clarithromycin; CR, case report; ITT, intention‐to‐treat; L, levofloxacin; M, metronidazole; Mi, minocycline; ND, not described; P, prospective; PP, per‐protocol; PPI, proton pump inhibitor; R, retrospective; RCT, randomized controlled trial; Rf, rifabutin; Si, sitafloxacin; T, tetracycline; V, vonoprazan.

^a^Susceptibility‐guided therapy.

**Table 3:** Quadruple therapy for *Helicobacter pylori* eradication in patients allergic to penicillin.

Author	Year	Country/region	Center	Study design	Sample size	Patient type	Treatment	Duration (days)	Eradication rate (ITT analysis, %)	Eradication rate (PP analysis, %)	Adverse reactions (%)	Compliance (%)
Gisbert JP et al.[13]	2005	Spain	M	P	17	2nd line	RBC + T + M	7	47	53	53	88

Tay CY et al.[42]	2012	Austrilia	S	P	69	2nd line	PPI + B + Rf + Cf^a^	10	94.2	94.2	/	/

Wu G et al.[43]	2013	China	S	P(RCT)	42	1st line	PPI + B + L + T	14	/	77.5	17.5	/
					42	1st line	PPI + B + L + C	14	/	76.2	19.0	/

Gisbert JP et al.[23]	2015	Spain	M	P	50	1st line	PPI + B + T + M	10	74	75	14	98
					24	2nd line	PPI + B + T + M	10	37	38	58	87
					3	3rd line	PPI + B + T + M	10	100	100	67	100

Katelaris PH et al.[44]	2015	Austrilia	S	P	23	Rescue	PPI + B + T + M	7	70.3	/	/	/

Li LH(2016)[45]	2016	China	S	P(RCT)	56	1st line	PPI + B + L + C	10	80.4	/	12.5	/
					55	1st line	PPI + B + L + Or	10	89.1	/	3.6	/

Long X et al.[46]	2018	China	S	P(RCT)	33	1st line	PPI + B + C + M	14	84.8	96.0	48.5	81.8

Yang XY et al.[47]	2018	China	S	P(RCT)	60	1st line	PPI + B + L + C	10	70	/	12	/
					60	1st line	PPI + B + L + Or	10	87	/	4	/

Gao W et al.[48]	2019	China	S	R	120	1st line	PPI + B + T + M	14	86.7	94.5	46.7	/

Song Z et al.[49]	2019	China	S	P	152	1st line	PPI + B + L + Ce	14	85.5	90.1	21.3	95.3

Luo L et al.[36]	2020	China	S	P	22	ND	PPI + B + C + M^a^	14	81.8	94.1	/	/
					10	ND	PPI + B + L + M^a^	14	80.0	100	/	/
					72	ND	PPI + B + T + M ^a^	14	97.2	100	/	/

Nyssen OP et al.[38]	2020	Europe	M	R	250	1st line	PPI + B + T + M	7‐14	91	92	29	96
					69	2nd line	PPI + B + T + M	7‐14	78	82	32	95
					18	3rd line	PPI + B + T + M	7‐14	78	78	39	94
					1	3rd line	PPI + C + M + L	7‐14	100	100	0	100

Zhou Y et al.[50]	2020	China	S	P	22	1st line	PPI + B + C + M	14	90.9	/	/	/

Chen SW et al.[51]	2020	China	S	P	41	1st line	PPI + B + T + M	14	68.3	/	7.3	/

Wu QN et al.[52]	2020	China	S	P	86	1st line	PPI + B + L + C	14	67.4	72.5	8.1	/
					86	1st line	PPI + B + L + Dx	14	84.0	88.0	11.6	/

Kong S et al.[53]	2021	China	S	CR	1	4th line	PPI + B + L + F	14	100	/	/	/

Zhang LY et al.[54]	2022	China	S	P(RCT)	74	1st line	PPI + B + Mi + L	14	89.2	90.4	38.8	/
					76	1st line	PPI + B + Mi + M	14	80.3	83.6	47.4	/

Qi T et al.[55]	2022	China	S	R	242	1st line	PPI + B + L + C	14	62.8	68.8	/	/

Zhao YQ et al.[56]	2022	China	S	P	43	1st line	PPI + B + Dx + F	14	93.1	95.2	9.3	/

Sun YC et al.[57]	2023	China	S	R	20	1st line	PPI + B + F + T (500 mg BID)	14	95	/	/	/
					28	1st line	PPI + B + F + T (500 mg TID)	14	89	/	/	/
					15	1st line	PPI + B + F + T (750 mg BID)	14	100	/	/	/
					4	2nd line	PPI + B + F + T (500 mg BID)	14	75	/	/	/
					2	2nd line	PPI + B + F + T (500 mg TID)	14	100	/	/	/
					6	2nd line	PPI + B + F + T (750 mg BID)	14	93	/	/	/

Zhang Y et al.[58]	2023	China	S	P(RCT)	150	1st line	PPI + B + Mi + Ce	14	82.7	90.9	22.6	91.8
					150	1st line	PPI + B + Ce + M	14	82.0	88.2	28.9	91.9
					150	1st line	PPI + B + Mi + M	14	84.0	91.7	35.1	90.5

Gao W et al.[43].	2024	China	S	P(RCT)	150	1st line	PPI + B + T + M	14	89.3	97.7	48.0	87.3

Wang H et al.[57]	2025	China	M	P(RCT)	124	1st line	V + B + Ce + T	14	90.3	92.4	21.8	96.8
					124	1st line	V + B + Ce + L	14	81.5	85.4	24.1	95.9

Yan TL et al.[42]	2024	China	M	P(RCT)	166	1st line	PPI + B + Dx + M	14	71.7	73.0	40.4	91.0
					166	1st line	V + B + Dx + M	14	90.4	92.6	39.8	88.0

Han Q, et al.[59]	2024	China	S	R	69	1st line	PPI + B + Mi + C	14	86.9	90.9	13.0	95.7
					71	1st line	PPI + B + C + M	14	60.5	66.2	16.9	91.5
					79	1st line	PPI + B + C + L	14	62.0	68.1	16.5	91.1

Lin MJ.[60]	2023	China	S	P(RCT)	31	1st line	V + B + C + L	14	87.1	94.1	19.4	96.8
	2023	China	S	P(RCT)	31	1st line	V + B + T + M	14	87.1	90.0	29.0	100.0
	2023	China	S	P	87	2nd line	V + B + T + F	14	89.7	98.7	/	/
	2023	China	S	P	35	3rd line	V + B + T + F	14	91.4	94.1	/	/
	2023	China	S	P	14	4th line	V + B + T + F	14	85.7	92.3	/	/

Wang XL et al.[61]	2025	China	S	P	80	ND	PPI + B + Ce + C	14	76.3	82.4	14.9	95.9
					80	ND	PPI + B + F + C	14	78.8	90.0	31.4	87.1

Abbreviations: B, bismuth; C, clarithromycin; Ce, cefuroxime; Cf, ciprofloxacin; CR, case report; Dx, doxycycline; ITT, intention‐to‐treat; L, levofloxacin; M, metronidazole; Mi, minocycline; ND, not described; Or, ornidazole; P, prospective; PP, per‐protocol; PPI, proton pump inhibitor; R, retrospective; RBC, ranitidine bismuth citrate; RCT, randomized controlled trial; Rf, rifabutin; Si, sitafloxacin; T, tetracycline; V, vonoprazan.

^a^Susceptibility‐guided therapy.

### 2.2. Risk‐of‐Bias and Quality Assessment

The methodological rigor of included studies was appraised using design‐specific tools. Randomized controlled trials (RCTs) were evaluated with the Cochrane Risk of Bias tool Version 2 (RoB 2), and observational cohort studies with the Newcastle‐Ottawa Scale (NOS). For other study designs (e.g., case report), their inherent methodological considerations are described narratively. The detailed, domain‐level judgments for included studies are provided in Tables S1, S2, S3.

## 3. Results

**Figure 1:**
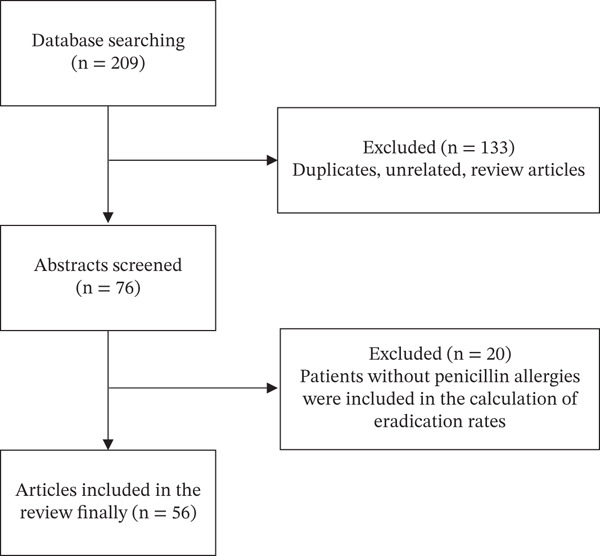
Flow of literature search.

Out of 209 publications identified, 56 studies were included in the review (93 were excluded as not relevant, 40 were review articles, and 20 were excluded because patients without penicillin allergies were included in the calculation of eradication rates). Figure 1 shows the process for article retrieval, screening, and inclusion. The final literature review comprised 132 treatment groups in 56 studies from nine different countries or regions, with a sample size of 5542 patients. Of these, 69 groups were primary treatment arms, 42 were rescue treatment arms, and 21 were not described.

Table 2 presents studies of triple therapy for *H. pylori* eradication in patients allergic to penicillin, whereas Table 3 shows studies of quadruple therapy. Table 4 displays the eradication rate of different regimens in patients allergic to penicillin.

**Table 4:** *Helicobacter pylori* eradication rate of different regimes in patients allergic to penicillin.

Regime		Patient type	Eradication rate (ITT analysis)	95% CI
Dual therapy	All regimes	Total^a^	92.7% (202/218)	89.2%–96.1%
	V + T	First‐line	92.9% (156/168)	88.9%–96.8%
		Rescue	90.9% (40/44)	82.1%–99.8%

Triple therapy	All regimes	Total	72.9% (1084/1486)	70.7%–75.2%
		First‐line	68.8% (660/960)	65.8%–71.7%
		Rescue	73.2% (197/269)	67.9%–78.6%
	PPI + C + L/PPI + C + M/PPI + L + M	Total	61.1% (518/848)	57.8%–64.4%
		First‐line	60.1% (407/677)	56.4%–63.8%
		Rescue	68.0% (87/128)	59.8%–76.2%
	PPI + T + M	Total	84.0% (21/25)	68.6%–99.4%
		First‐line	81.8% (18/22)	64.3%–99.3%
		Rescue	100% (3/3)	100%–100%
	Triple regimen containing sitafloxacin (PPI + Si + M/V + Si + M)	Total	87.9% (304/346)	84.4%–91.3%
		First‐line	86.2% (144/167)	80.9%–91.5%
		Rescue	87.1% (101/116)	80.9%–93.3%
	Triple regimen containing vonoprazan	Total	94.1% (222/236)	91.0%–97.1%
		First‐line	96.1% (99/103)	92.3%–99.9%
		Rescue	76.9% (20/26)	59.6%–94.3%

Quadruple therapy	All regimes	Total	90.9% (3112/3838)	82.1%–99.8%
		First‐line	80.8% (2586/3201)	79.4%–82.2%
		Rescue	82.0% (306/373)	78.1%–86.0%
	PPI + B + T + M	Total	81.4% (609/748)	78.6%–84.2%
		First‐line	83.5% (510/611)	80.5%–86.4%
		Rescue	72.3% (99/137)	64.7%–79.9%
	PPI + B + C + L/PPI + B + C + M/PPI + B + L + M	First‐line^b^	70.7% (570/806)	67.6%–73.9%
	Quadruple regimen containing semisynthetic tetracycline (PPI/V + B + Mi/Dx + L/M/F)	First‐line^b^	83.5% (818/980)	81.1%–85.8%
	Quadruple regimen containing cefuroxime (PPI/V + B + Ce + L/M/Mi)	First‐line^b^	84.1% (729/867)	81.6%–86.5%
	Quadruple regimen containing vonoprazan	Total	88.1% (539/612)	85.5%–90.6%
		First‐line	87.6% (417/476)	84.6%–90.6%
		Rescue	90.4% (122/136)	85.4%–95.4%

Abbreviations: B, bismuth; C, clarithromycin; Ce, cefuroxime; CI, confidence interval; Dx, doxycycline; ITT, intention‐to‐treat; L, levofloxacin; M, metronidazole; Mi, minocycline; PPI, proton pump inhibitor; Si, sitafloxacin; T, tetracycline; V, vonoprazan.

^a^Total groups include treatment‐naive groups, treatment‐experienced groups, and groups with no classification of treatment‐naive or ‐experienced.

^b^No rescue treatment studies.

### 3.1. Triple Therapy

#### 3.1.1. PPI in Combination With Two of the Following Antibiotics: Clarithromycin, Levofloxacin, and Metronidazole

Most studies used this triple regimen due to the worldwide availability of clarithromycin, levofloxacin, and metronidazole. Eradication therapy was conducted for 848 penicillin‐allergic patients with *H. pylori* infection across 23 treatment groups in 13 studies, including 12 treatment‐naive groups, nine treatment‐experienced groups, and two groups with no classification of treatment‐naive or ‐experienced. However, the overall eradication efficacy was unacceptable, with eradication rates of 61.1% (95% CI 57.8%–64.4%) in ITT analysis [13, 17, 18, 21, 23, 30, 32–35, 38, 40, 41]. This may be related to the generally high resistance of local subjects to these antibiotics.

#### 3.1.2. PPI–Tetracycline‐Metronidazole Regimen

Three treatment groups in two studies used this regimen for eradication therapy, with two first‐line treatment groups and one treatment‐experienced group. A total of 25 patients were included. This triple therapy showed a mean eradication rate of 84.0% (21/25, 95% CI 68.6%–99.4%) in ITT analysis, and an eradication rate of 100% in PP analysis [14, 15]. However, both the number of studies and sample size were minimal. Some studies chose to include a bismuth compound to further improve eradication efficacy (see quadruple therapy for details).

#### 3.1.3. Triple Regimen Containing Sitafloxacin

Ten studies conducted in Japan investigated the triple therapy regimen containing sitafloxacin, comprising 24 treatment groups, including nine first‐line treatment groups, 11 treatment‐experienced groups, and four groups without treatment classification, involving 346 patients. This regimen demonstrated an acceptable mean eradication efficacy of 87.9% (95% CI 84.4%–91.3%) [19–21, 25, 27–30, 32, 33, 37, 39, 41]. However, sitafloxacin is currently only available in Japan, suggesting that its high efficacy may not be broadly applicable to other regions.

#### 3.1.4. Triple Regimen Containing Vonoprazan

Seven trials, comprising 16 treatment groups (six first‐line, five rescue, and five undefined), used a triple regimen with vonoprazan for *H. pylori* eradication in 236 penicillin‐allergic patients [24, 27, 30, 32, 33, 39, 41]. All seven studies reported good eradication results, with a mean eradication rate of 94.1% (95% CI 91.0.7%–97.1%). However, triple‐drug regimens containing vonoprazan are primarily used in regions with low antibiotic resistance, such as Japan, due to differing antibiotic resistance profiles. In areas with high resistance, dual‐ and quadruple‐drug regimens containing vonoprazan are more commonly used.

#### 3.1.5. Other Triple Regimens

Several studies investigated the eradication efficacy of triple therapy regimens containing minocycline (three studies, four treatment groups, 32 patients) [16, 24, 31], rifabutin (two studies, three treatment groups, 18 patients) [13, 23], or individualized treatment (three studies, seven treatment groups, 108 patients) [22, 26, 36]. Although the overall eradication efficacy is acceptable (85.7% in ITT analysis, 95% CI 79.8%–91.6%), the limited number and size of these studies warrant further research to draw definitive conclusions.

The overall eradication efficacy of these triple therapies remains unacceptable. Despite studies on new drugs, regimens, and combinations, the limited quantity and quality of these studies makes the efficacy of these regimens uncertain.

### 3.2. Quadruple Therapy

#### 3.2.1. PPI–Bismuth–Tetracycline–Metronidazole Regimen

The classic BQT stands as the most prevalent eradication regimen recommended for patients with a penicillin allergy in various diagnostic and treatment guidelines. This therapy was studied across six research papers encompassing 10 treatment groups, which included five treatment‐naive groups and five treatment‐experienced groups, involving a total of 748 patients. Although reported eradication rates varied among these studies, the overall efficacy was deemed satisfactory, with eradication rates of 81.4% (95% CI 78.6%–84.2%) in ITT analysis [13, 23, 38, 43, 44, 48, 51]. The classical BQT is supported by the largest number of studies, the highest total sample size, and the most widely recognized efficacy. However, this regimen is often limited in clinical use due to the high incidence of adverse reactions, poor patient tolerance, limited availability of tetracycline and bismuth in many regions, and the relatively cumbersome and complex dosing.

#### 3.2.2. Combination of PPI‐Bismuth and Two of the Following Antibiotics: Clarithromycin, Metronidazole, or Levofloxacin

Eight studies included 11 treatment groups, all treatment‐naive, comprising 806 patients using a quadruple regimen of PPI‐bismuth and two antibiotics, including clarithromycin, levofloxacin, or metronidazole, for penicillin‐allergic patients. The overall eradication efficacy was not well, with rates of 70.7% (95% CI 67.6%–73.9%) in ITT analysis. This may be due to the generally high resistance of local subjects to clarithromycin, levofloxacin, or metronidazole [34, 45, 47, 50, 54, 55, 56].

#### 3.2.3. Quadruple Regimen Containing Semisynthetic Tetracycline

Six studies [42, 49, 50, 53, 57, 58] with nine first‐line treatment groups and 980 patients used semisynthetic tetracyclines such as minocycline or doxycycline for eradication therapy, with good overall efficacy showing eradication rates of 83.5% (95% CI 81.1%–85.8%) in ITT analysis, as well as a good safety profile and good patient compliance. When classical tetracyclines are difficult to obtain, semisynthetic tetracyclines can be considered as an alternative.

#### 3.2.4. Quadruple Regimen Containing Cefuroxime (Second‐Generation Cephalosporin)

Five studies [49, 53, 56, 60, 61] included seven first‐line treatment groups and 867 patients using a quadruple regimen containing cefuroxime to eradicate *H. pylori* in people with penicillin allergy. The overall eradication efficacy was good, with rates of 84.1% (95% CI 81.6%–86.5%) in ITT analysis. The safety profile and compliance were also good. No significant cross‐allergic reactions with penicillin were observed, indicating a good prospect for practical application. However, the number of studies was limited, and all are from China, with data lacking from other regions.

#### 3.2.5. Quadruple Regimen Containing Vonoprazan

Three studies included five first‐line treatment groups and three rescue treatment groups including 612 patients using a quadruple regimen containing vonoprazan to eradicate *H. pylori* in people with penicillin allergy [42, 56, 59]. The overall eradication efficacy was good, with rates of 88.1% (95% CI 85.5%–90.6%) in ITT analysis. With the increasing availability of vonoprazan in regions with high antibiotic resistance, such as China, the application of quadruple eradication regimens containing vonoprazan has expanded significantly and achieved favorable outcomes.

#### 3.2.6. Other Regimens

A few studies have explored alternative eradication options, including individualized treatment, modified concomitant regimens (such as the PPI‐clarithromycin–metronidazole–levofloxacin regimen), quadruple regimens containing a combination of levofloxacin and tetracycline, quadruple regimens containing ranitidine bismuth citrate, and quadruple regimens containing rare antibiotics like rifabutin or furazolidone. These studies, involving 389 patients, generally reported good eradication efficacy (85.9% in ITT analysis, 95% CI 82.4%–89.3%) [13, 36, 38, 46, 62–64]. However, due to the limited number of studies, small sample sizes of each study, inconsistent eradication efficacy, and high heterogeneity, further research is necessary to validate these findings [42].

Classical BQT is currently the most recommended quadruple regimen, supported by relatively reliable research and efficacy. The application of quadruple eradication regimens containing vonoprazan has gradually expanded and achieved favorable outcomes. Semisynthetic tetracyclines can be a promising alternative when classical tetracyclines are not readily available. Substituting amoxicillin with cefuroxime is also a potential strategy to improve the eradication rate. Limited research evidence is available for other new combinations and regimens, necessitating further investigation to confirm their efficacy.

### 3.3. Dual Therapy

Four studies, comprising five treatment groups (including four first‐line treatment groups and one treatment‐experienced group), involving 218 patients, used a dual drug regimen of a PPI or potassium‐competitive acid blocker (P‐CAB) combined with an antibiotic for eradication therapy in patients with a penicillin allergy [65–67].

Historically, two studies involving six patients used the dual therapy of PPI‐clarithromycin, demonstrating acceptable eradication efficacy [65, 66]. However, due to the current high rates of clarithromycin resistance, this therapy is not recommended.

More recently, some researchers employed the dual therapy of vonoprazan‐tetracycline (VPZ–TET )for eradication therapy in 212 patients with a penicillin allergy [43, 66]. Both first‐ and second‐line treatments in ITT analysis showed an eradication rate above 90%. Nevertheless, more clinical studies in different countries are necessary to further confirm the efficacy of this regimen.

## 4. New Regimens to Improve Eradication Efficacy

### 4.1. Minocycline‐Containing Regimen

Minocycline is a semisynthetic tetracycline that acts on bacterial ribosomes, inhibiting protein synthesis [68]. Compared with tetracycline, minocycline has a higher absorption rate, is less influenced by food, and has a longer half‐life, requiring only once or twice daily dosing, which enhances patient compliance [69, 70]. In the treatment of other infectious diseases, minocycline has demonstrated superior eradication efficacy and safety compared with tetracycline and is widely used in clinical practice. In vitro antibiotic susceptibility testing has shown that minocycline exhibits good activity against *H. pylori* with a low resistance rate, similar to tetracycline [5].

Suo et al. conducted a comparative study evaluating the efficacy, safety, and compliance of tetracycline‐containing and minocycline‐containing quadruple regimens in patients without a penicillin allergy. Both regimens showed similar results, suggesting that minocycline could be an effective alternative to tetracycline in areas where tetracycline is scarce for *H. pylori* eradication therapy [71].

In a randomized controlled trial conducted by our center in treatment‐naive patients with a penicillin allergy and *H. pylori* infection, both the quadruple regimen containing minocycline (100 mg, twice daily)–metronidazole (400 mg, four times daily) (*n* = 150, with an eradication rate of 84% in ITT analysis, 91.7% in PP analysis) and the quadruple regimen containing minocycline (100 mg, twice daily)–cefuroxime (500 mg, twice daily) (*n* = 150, with an eradication rate of 82.7% in ITT analysis, 90.9% in PP analysis) achieved good eradication efficacy, safety, and compliance [49].

In another randomized controlled trial by Zhang YL et al., comparing the eradication efficacy of the quadruple regimen containing minocycline (100 mg, twice daily) and levofloxacin (400 mg, once daily) with a quadruple regimen containing minocycline (100 mg, twice daily) and metronidazole (400 mg, three times daily) in treatment‐naive penicillin‐allergic patients with *H. pylori* infection, the results showed that the quadruple regimen containing minocycline‐levofloxacin was superior to that containing minocycline–metronidazole (eradication rate 89.2% vs. 80.3% in ITT analysis, 90.4% vs. 83.6% in PP analysis) [57]. Both studies indicate that the minocycline‐containing regimen has better eradication efficacy in patients with penicillin allergy.

In conclusion, minocycline maintains exceptionally low and stable resistance rates across most regions and serves as a cost‐effective alternative due to its superior accessibility compared with tetracycline. Its primary disadvantage is the frequent occurrence of vestibular adverse events, such as dizziness, which may compromise patient compliance during a full therapeutic course. Further research is needed to confirm its effectiveness and safety.

### 4.2. Cephalosporins‐Containing Regimen

Cephalosporins and amoxicillin are both *β*‐lactam antibiotics that exert their bactericidal effects by inhibiting cell wall synthesis [72, 73]. In vitro drug susceptibility tests have shown that *H. pylori* strains are sensitive to cephalosporins [5]. In clinical application, cephalosporins are widely available in most hospitals, do not require skin testing before oral administration, are highly stable in the presence of gastric acid, and have high bioavailability [74]. Although there is a 10% cross‐allergy between cephalosporins and amoxicillin, this primarily occurs with first‐generation cephalosporins that have a side‐chain structure similar to penicillin and rarely occurs with second‐ and third‐generation cephalosporins [74–77].

A randomized controlled trial at our center used a second‐generation cephalosporin, cefuroxime, instead of amoxicillin for the eradication of *H. pylori* infection in patients without a penicillin allergy and achieved satisfactory eradication efficacy similar to that of amoxicillin [78]. In another prospective cohort study conducted in treatment‐naive patients (*n* = 152) with a penicillin allergy, the quadruple regimen containing cefuroxime and levofloxacin showed satisfactory eradication efficacy (eradication rate of 85.5% in ITT analysis, 90.1% in PP analysis), safety (adverse reaction incidence rate of 21.3%), and compliance (95.2%) [52]. In a recently published three‐arm RCT study, the quadruple regimen containing minocycline–cefuroxime and the quadruple regimen containing cefuroxime‐metronidazole also showed excellent eradication efficacy in patients with a penicillin allergy (eradication rates of 82% and 82.7% in ITT analysis, 88.2% and 90.9% in PP analysis) [49]. No severe cross‐allergy occurred. These studies suggest that cefuroxime has the potential to replace amoxicillin in the eradication of *H. pylori* in patients allergic to penicillin.

### 4.3. Rifabutin‐Containing Regimen

Rifabutin is a semisynthetic rifamycin that inhibits DNA‐dependent RNA polymerase in bacteria, disrupting the transcription process. The low rate of resistance of *H. pylori* to rifabutin (~0.13%) suggests that rifabutin may be a new option for eradication therapy [79]. In published studies, rifabutin is primarily used for treatment‐experienced patients. Gisbert conducted a systematic review of 21 studies of rescue therapy with rifabutin‐containing regimens and found that the overall ITT eradication success rate was 73%, with a rate of 79% for second‐line regimens and 66%–70% for third‐ or greater line regimens, suggesting that rifabutin can be used as rescue therapy after multiple eradication failures [79].

A small number of studies have also used rifabutin for rescue treatment of *H. pylori* infection in penicillin‐allergic patients, but all have small sample sizes, large variations in eradication rates, and need to be supported by further clinical trials [13, 23, 62]. In addition, some safety concerns remain with a rifabutin‐containing regimen, which carries a risk of severe side effects such as myelotoxicity. Furthermore, some researchers have suggested a risk of inducing resistance in Mycobacterium tuberculosis by the widespread use of rifabutin in *H. pylori* eradication therapy in areas of high tuberculosis prevalence. Further research is needed.

### 4.4. Sitafloxacin‐Containing Regimen

Sitafloxacin is a fourth‐generation fluoroquinolone antibiotic that inhibits DNA helicase activity, thereby hindering genetic material replication and exerting bactericidal effects. Fluoroquinolone resistance in *H. pylori* is mainly due to Gyr A mutations. Sitafloxacin stands out among fluoroquinolones as it effectively targets Gyr A mutant *H. pylori* strains, reducing the likelihood of resistance development during treatment and enhancing eradication efficacy [29]. In patients allergic to penicillin, 10 studies (comprising 24 treatment groups, nine first‐line treatment groups, 11 treatment‐experienced groups, and four groups without treatment classification, totaling 346 patients) have employed the sitafloxacin‐containing regimen for *H. pylori* eradication therapy. These studies have consistently shown good eradication efficacy, with most achieving eradication rates (ITT or PP analysis) exceeding 90%. However, all studies were restricted to Japan. Moreover, the number and sample size of studies are relatively small, and all have used the triple regimen containing metronidazole, which has higher resistance rates in other countries.

In summary, sitafloxacin exhibits potent bactericidal activity against multidrug‐resistant strains, making it a high‐efficacy option for rescue therapy in penicillin‐allergic patients. Nevertheless, its clinical utility is hindered by very limited global accessibility—primarily restricted to Japan—alongside higher costs and potential fluoroquinolone‐related adverse effects. Furthermore, the low resistance rate of *H. pylori* to quinolones or metronidazole in Japan differs from other regions where these antibiotics are. Therefore, the efficacy of sitafloxacin for eradicating *H. pylori* in patients with penicillin allergy needs further evaluation through larger studies in diverse regions.

### 4.5. Vonoprazan‐Containing Regimen

Vonoprazan is a novel P‐CAB with potent acid‐suppressive effects, rapid onset of action, metabolism independent of the CYP2C19 genotype, and prolonged acid‐suppressive duration compared with conventional PPIs [80]. Several studies from Japan have employed a combination of vonoprazan and two antibiotics (clarithromycin, metronidazole, or minocycline) for treating *H. pylori* infection in penicillin‐allergic patients, yielding promising eradication rates. For first‐line treatments, the eradication rates were 96.1% (95% CI 92.3%–99.9%) (four studies, six treatment groups, 103 patients), whereas for rescue treatments, the rates were 76.9% (95% CI 59.6%–94.3%) (three studies, five treatment groups, 26 patients) [30, 32, 39].

Recently, clinical trials have increasingly supported the efficacy of P‐CAB‐based dual therapies as viable alternatives to traditional, more complex quadruple regimens. Gao et al. [43, 67] evaluated VPZ–TET dual therapy in penicillin‐allergic patients. Their exploratory real‐world study (2023) and subsequent randomized controlled trial both demonstrated ITT eradication rates exceeding 90%. The consistently elevated intragastric pH (> 6.0) achieved by vonoprazan is believed to enhance the antimicrobial efficacy of tetracycline, supporting the use of this streamlined regimen that offers reduced pill burden and improved patient adherence.

In more complex clinical scenarios—such as rescue therapy or regions with high antibiotic resistance—P‐CABs have also been incorporated into BQT protocols. Yan et al. [42] reported that a vonoprazan‐based BQT regimen containing doxycycline and metronidazole significantly outperformed a traditional PPI‐based BQT in penicillin‐allergic patients (ITT eradication rate: 92.4% vs. 84.1%). This finding highlights the potential of P‐CABs to enhance eradication efficacy even in settings where antibiotic resistance may compromise standard treatments.

Furthermore, Wang et al. [56] explored a novel bismuth quadruple regimen incorporating cefuroxime and tetracycline. Their results underscored the importance of a stable acidic environment for maximizing the effectiveness of cephalosporins, particularly in patients with penicillin allergy.

In summary, vonoprazan has been demonstrated to enhance the efficacy of nonamoxicillin regimens, such as those containing tetracyclines, through potent and sustained acid suppression independent from the CYP2C19 polymorphism, whereas simplified dual dosing schedules have been shown to significantly improve patient compliance. As becoming available in more areas, the use of vonoprazan holds promise as a means to enhance *H. pylori* eradication efficacy in patients allergic to penicillin. Nevertheless, the cost‐effectiveness of the latter remains to be ascertained, and its accessibility is still limited in certain regions and primary care settings.

### 4.6. Individualized Treatment

Increased antibiotic resistance significantly impacts the eradication rate of *H. pylori* infection. Some studies have implemented *H. pylori* culture and drug‐sensitivity testing prior to treatment. Patients received personalized treatment based on the drug‐sensitivity results, achieving an ITT eradication rate of 93.3% (95% CI 89.7%–96.8%) [22, 26, 36]. This indicates that drug sensitivity‐guided individualized treatment could be effective for penicillin‐allergic patients. However, challenges exist in the widespread application of this sensitivity‐guided strategy in clinical settings: drug‐sensitivity testing relies on gastroscopic biopsy sampling and *H. pylori* strain culture, which are invasive, time‐consuming, and costly, limiting the adoption of individualized treatment [81].

In clinical practice, tetracycline resistance is extremely rare. Although metronidazole has a high resistance rate in many areas, the combination of a quadruple regimen by extending the course of treatment, increasing the dose of metronidazole, and pairing it with bismuth can be effective in overcoming metronidazole resistance. Therefore, in areas where tetracycline and bismuth are available, empirical use of the classical bismuth quadruple regimen is more cost‐effective and practical, given the cost‐effectiveness and convenience of not requiring invasive testing. In contrast, in areas where tetracycline and bismuth are difficult to obtain, or where clarithromycin and quinolone resistance is high, there is still a lack of effective empirical treatment protocols. Individualized therapy may be a more effective treatment approach in these cases [82].

## 5. Reevaluation of Penicillin Allergy

Many patients exhibit false–positive penicillin skin tests. Factors contributing to false–positive results include the absence of a negative control for the penicillin skin test, impurities in the skin test drug, nonstandardized skin test procedures, and loose interpretation of skin test results. In some countries, outpatient clinics lack options for testing, and the assessment of penicillin allergy relies on patient recollection, lacking objective assessment criteria. Several studies have demonstrated that 80% of patients who reported penicillin allergy had negative skin testing and could safely receive penicillin therapy. A patient′s history of penicillin allergy should be approached with caution, and a comprehensive and accurate allergy history, including description and timing of adverse reactions, should be obtained. Reevaluation for the presence of penicillin allergy may be necessary. Standardization of the skin test procedure and interpretation of results is crucial. In cases of false–positive results, treatment with amoxicillin‐containing regimens may be acceptable under close observation after reevaluation [10].

## 6. Conclusion

*H. pylori* eradication in patients with penicillin allergy presents a significant challenge. Presently, the efficacy of triple therapy regimens is generally unacceptable. The classical BQT stands as the most commonly prescribed regimen due to its well‐documented efficacy in the literature. In recent years, P‐CABs have become a cornerstone in the treatment of *H. pylori* infection among penicillin‐allergic patients. Whether employed in simplified dual regimens or in bismuth‐based quadruple therapies, P‐CAB–centered strategies offer high‐eradication rates, favorable tolerability, and a promising direction toward standardized, effective treatment approaches for this challenging subgroup. More novel drugs and regimens, such as minocycline, cefuroxime, sitafloxacin, and rifabutin, have emerged, showing promise but requiring further confirmation of efficacy. Individualized treatment may be a promising direction in the future, incorporating rapid and simple testing for drug‐resistant gene mutations. Standardized evaluation for penicillin allergy is essential to avoid false–positive results. Clinicians should select the optimal treatment regimen based on local drug resistance patterns, drug availability, and patient medical conditions. However, many studies come from certain countries such as Japan and China, and the current body of literature on *H*.*p* *y* *l* *o* *r* *i* eradication in penicillin‐allergic patients is limited in quantity and quality, with relatively small sample sizes, making it difficult to perform a high‐quality meta‐analysis. More high‐quality, large‐scale clinical studies are warranted to determine the most effective eradication therapy regimen.

## Author Contributions

Yuxin Zhang and Baojun Suo drafted the initial manuscript. Xueli Tian, Cailing Li, Xinlu Ren, Kai Zhou, Changmin Mi, Yuxin Wang, and Lingling Ma conducted the literature search. Liya Zhou revised the manuscript. Zhiqiang Song supervised the study and edited the manuscript. All authors contributed to the article. Yuxin Zhang and Baojun Suo contributed equally to this work.

## Funding

This study was supported by Foundation for Innovative Research Groups of the National Natural Science Foundation of China (10.13039/501100001809, No. 82170562); the Beijing Natural Science Foundation (No. 7232199); the Capital′s Funds for Health Improvement and Research (No. 2022‐2‐4093); the Youth Incubation Fund of Peking University Third Hospital (No. BYSYFY2021003); the Key Laboratory for *Helicobacter pylori* Infection and Upper Gastrointestinal Diseases in Beijing (No. BZ0371).

## Disclosure

All authors approved the final version for submission. The funding sources had no role in the study design, data collection and analysis, decision to publish, or preparation of the manuscript.

## Conflicts of Interest

The authors declare no conflicts of interest.

## Supporting Information

Additional supporting information can be found online in the Supporting Information section.

## Supporting information


**Supporting Information 1** Table S1 presents the detailed quality assessment of included randomized controlled trials using the RoB 2 tool.

**Supporting Information 2** Table S2 details the quality assessment of included cohort studies using the Newcastle‐Ottawa Scale (NOS).

**Supporting Information 3** Table S3 provides the quality assessment results for other included studies.

## Data Availability

The data that support the findings of this study are available from the corresponding author upon reasonable request.
