# Supplementary material for: A Review on the Progress and Strategies of Helicobacter pylori Eradication Therapy for Patients With Penicillin Allergy
Source: Gastroenterol Res Pract. 2026 Apr 8;2026:5587248. doi: 10.1155/grp/5587248 (PMC13062287; doi:10.1155/grp/5587248)
Supplement: Supplementary file 1 — Supporting Information 1 Table S1 presents the detailed quality assessment of included randomized controlled trials using the RoB 2 tool. [file GRP-2026-5587248-s001.docx]

**Supplementary Table 1. Summary of Risk of Bias Assessment for Randomized controlled trial with ROB2**

| **Study ID** | **Study**  **Design** | **Randomization process** | **Deviations from intended interventions** | **Missing outcome data** | **Measurement of the outcome** | **Selection of the reported result** | **Overall Risk** |
| --- | --- | --- | --- | --- | --- | --- | --- |
| Prach 1998 | RCT | Some concerns | Some concerns | Low | Low | Low | Some concerns |
| Tavakoli 1999 | RCT | Some concerns | Some concerns | Low | Low | Low | Some concerns |
| Li 2016 | RCT | Some concerns | Some concerns | Low risks | Low risks | Some concerns | Some concerns |
| Long 2018 | RCT | Low | Some concerns | Low | Low | Low | Low |
| Yang 2018 | RCT | Some concerns | Some concerns | Low risks | Low risks | Some concerns | Some concerns |
| Zhang 2022 | RCT | Low | Some concerns | Low | Low | Some concerns | Some concerns |
| Chen 2020 | RCT | Some concerns | Some concerns | Low risks | Low risks | Some concerns | Some concerns |
| Zhang 2023 | RCT | Low | Some concerns | Low | Low | Low | Low |
| Lin 2023 | RCT | Low | Some concerns | Low | Low | Low | Low |
| Gao 2024 | RCT | Low | Some concerns | Low | Low | Low | Low |
| Wang 2025 | RCT | Low | Some concerns | Low | Low | Low | Low |

RCT: Randomized controlled trial；ROB:
