# Supplementary material for: A Review on the Progress and Strategies of Helicobacter pylori Eradication Therapy for Patients With Penicillin Allergy
Source: Gastroenterol Res Pract. 2026 Apr 8;2026:5587248. doi: 10.1155/grp/5587248 (PMC13062287; doi:10.1155/grp/5587248)
Supplement: Supplementary file 2 — Supporting Information 2 Table S2 details the quality assessment of included cohort studies using the Newcastle‐Ottawa Scale (NOS). [file GRP-2026-5587248-s002.docx]

**Supplementary Table 2. Summary of Risk of Bias Assessment for Cohort Studies with NOS**

| **Study** | **Design** | **Selection** | **Comparability** | **Outcome** | **Tota** | **Quality** |
| --- | --- | --- | --- | --- | --- | --- |
| Gisbert 2005 | P | 3 | 0 | 3 | 6 | Moderate |
| Rodríguez-Torres 2005 | P | 3 | 0 | 3 | 6 | Moderate |
| Matsushima 2006 | R | 2 | 2 | 2 | 6 | Moderate |
| Gisbert 2010 | P | 4 | 1 | 3 | 8 | High |
| Tay 2012 | P | 3 | 2 | 2 | 7 | High |
| Gisbert 2015 | P | 3 | 2 | 3 | 8 | High |
| Ono 2017 | R | 2 | 2 | 3 | 7 | High |
| Tanaka 2017 | P | 3 | 2 | 3 | 8 | High |
| Ono 2017 | R | 3 | 1 | 2 | 6 | Moderate |
| Sue 2017 | P+R | 4 | 2 | 3 | 9 | High |
| Long 2018 | P | 3 | 2 | 3 | 8 | High |
| Song 2019 | P | 3 | 2 | 3 | 8 | High |
| Zhang 2019 | R | 2 | 1 | 1 | 4 | Moderate |
| Song 2019 | P | 4 | 2 | 3 | 9 | High |
| Gao 2019 | R | 2 | 1 | 3 | 6 | Moderate |
| Tepes 2020 | P | 3 | 2 | 3 | 8 | High |
| Masaoka 2020 | P | 3 | 2 | 2 | 7 | High |
| Zhou 2020 | P | 3 | 2 | 3 | 8 | High |
| Luo 2020 | P | 3 | 0 | 3 | 6 | Moderate |
| Nyssen 2020 | P | 4 | 2 | 3 | 9 | High |
| Wu 2020 | R | 2 | 1 | 2 | 5 | Moderate |
| Sue 2021 | P | 3 | 0 | 2 | 5 | Moderate |
| Qi 2022 | R | 3 | 1 | 2 | 6 | Moderate |
| Zhao 2022 | P | 3 | 1 | 3 | 7 | High |
| Adachi 2023 | P | 3 | 2 | 3 | 8 | High |
| Gao 2023 | R | 2 | 1 | 3 | 6 | Moderate |
| Sun 2023 | R | 4 | 2 | 2 | 8 | High |
| Han 2024 | R | 3 | 0 | 3 | 6 | Moderate |
| Zhang 2024 | P | 3 | 0 | 3 | 6 | Moderate |
| Wang 2025 | P | 3 | 1 | 3 | 7 | High |
| Mori 2017 | P | 3 | 2 | 3 | 9 | High |

P, prospective cohort study; R, retrospective cohort study; NOS, Newcastle - Ottawa quality assessment scale.
