# Supplementary material for: A Review on the Progress and Strategies of Helicobacter pylori Eradication Therapy for Patients With Penicillin Allergy
Source: Gastroenterol Res Pract. 2026 Apr 8;2026:5587248. doi: 10.1155/grp/5587248 (PMC13062287; doi:10.1155/grp/5587248)
Supplement: Supplementary file 3 — Supporting Information 3 Table S3 provides the quality assessment results for other included studies. [file GRP-2026-5587248-s003.docx]

**Supplementary Table 3. Summary of Risk of Bias Assessment for Other Studies**

| **Study** | **Design** | **Assessment tool** | **Quality** |  |
| --- | --- | --- | --- | --- |
| Konno 2010 | Case report | JBI | High |  |
| Kong 2021 | Case report | JBI | High |  |
| Furuta 2014 | Single-arm Clinical Study | JBI | Moderate |  |
| Osumi 2017 | Retrospective Diagnostic Study | JBI | High |  |
| Wu 2013 | RCT (Conference Abstract) | Unable to Assess | Limited Details |  |
| Siala 2010 | Retrospective Cohort Study (Conference Abstract) | Unable to Assess | Limited Details |  |
| Sahara 2013 | Single-arm Clinical Study (Conference Abstract) | Unable to Assess | Limited Details |  |
| Tanaka 2014 | Non-Randomized, Intervention Study (Conference Abstract) | Unable to Assess | Limited Details |  |
| Katelaris 2015 | Prospective Cohort Study (Conference Abstract) | Unable to Assess | Limited Details |  |
| Furuta 2015 | Single-Arm Clinical Study (Conference Abstract) | Unable to Assess | Limited Details |  |
| Kataoka 2016 | Prospective Cohort Study (Conference Abstract) | Unable to Assess | Limited Details |  |
| Tanaka 2016 | Prospective Cohort Study (Conference Abstract) | Unable to Assess | Limited Details |  |
| Tanaka 2017 | Retrospective Cohort Study (Conference Abstract) | Unable to Assess | Limited Details |  |
| Furuta 2017 | Retrospective Cohort Study (Conference Abstract) | Unable to Assess | Limited details |  |
